# Enhanced Late Na and Ca Currents as Effective Antiarrhythmic Drug Targets

**DOI:** 10.3389/fphar.2017.00036

**Published:** 2017-02-06

**Authors:** Hrayr S. Karagueuzian, Arash Pezhouman, Marina Angelini, Riccardo Olcese

**Affiliations:** ^1^From the Translational Arrhythmia Section, David Geffen School of Medicine, University of California, Los AngelesLos Angeles, CA, USA; ^2^Cardiovascular Research Laboratory, Departments of Medicine (Cardiology), David Geffen School of Medicine, University of California, Los AngelesLos Angeles, CA, USA; ^3^Department of Anesthesiology and Perioperative Medicine, David Geffen School of Medicine, University of California, Los AngelesLos Angeles, CA, USA; ^4^Department of Physiology, David Geffen School of Medicine, University of California, Los AngelesLos Angeles, CA, USA

**Keywords:** late Na current, late Ca current, ventricular fibrillation, early afterdepolarization, triggered activity, roscovitine, GS-458-967, antiarrhythmic drugs

## Abstract

While recent advances clarified the molecular and cellular modes of action of antiarrhythmic drugs (AADs), their link to suppression of dynamical arrhythmia mechanisms remains only partially understood. The current classifications of AADs (Classes I, III, and IV) rely on blocking peak Na, K and L-type calcium currents (I_Ca,L_), with Class II with dominant beta receptor blocking activity and Class V including drugs with diverse classes of actions. The discovery that the calcium and redox sensor, cardiac Ca/calmodulin-dependent protein kinase II (CaMKII) enhances both the late Na (I_Na-L_) and the late I_Ca,L_ in patients at high risk of VT/VF provided a new and a rational AAD target. Pathological rise of either or both of I_Na-L_ and late I_Ca,L_ are demonstrated to promote cellular early afterdepolarizations (EADs) and EAD-mediated triggered activity that can initiate VT/VF in remodeled hearts. Selective inhibition of the I_Na-L_ without affecting their peak transients with the highly specific prototype drug, GS-967 suppresses these EAD-mediated VT/VFs. As in the case of I_Na-L_, selective inhibition of the late I_Ca,L_ without affecting its peak with the prototype drug, roscovitine suppressed oxidative EAD-mediated VT/VF. These findings indicate that specific blockers of the late inward currents without affecting their peaks (gating modifiers), offer a new and effective AAD class action i.e., “Class VI.” The development of safe drugs with selective Class VI actions provides a rational and effective approach to treat VT/VF particularly in cardiac conditions associated with enhanced CaMKII activity such as heart failure.

## A brief historical perspective of antiarrhythmic drug (AAD) therapy

It may seem ironic that “antiarrhythmic” drug therapy long antedated the electrocardiographic diagnosis of cardiac arrhythmias when the French physician Jean Baptist de Sénac first described in 1749 the benefits of quinine in patients with “palpitations,” an implicit reference to atrial fibrillation (AF).

*“Of all the stomachic remedies the one whose effects appeared to be the most constant and the most prompt in many cases is quinine, mixed with a little rhubarb. Long and rebellious palpitations have ceded to this febrifuge seconded with a light purgative” (Willius and Keys, 1941)*.

The Paris-based physician, who perhaps was the first to describe in patients the correlation between palpitation (AF) and mitral valve disease, did extensive research on the cinchona extract that contains quinine/quinidine and the plant rhubarb that contains anthraquinones (laxatives). Sénac however, did not know if the fever reducing effect of quinine or the laxative effect of the anthraquinones or their combination were responsible to halt the presumed AF (Karagueuzian, 1995). Sénac's dilemma was apparently solved more than 150 years later thanks to a Dutch merchant who had discovered for himself the value of quinine against AF. The traveling merchant, who regularly took quinine to prevent malaria during his trips to the West Indies suffered from AF and was a patient of no less than the famed cardiologist Wenckebach, who recalled the experience of his patient this way:

*“In 1912, a patient presented himself in my office wishing to get rid of his attacks of auricular fibrillation…On my telling him that I could promise nothing, he told me that he knew how to get rid of his attacks, as I did not believe him he promised to come back the next morning with a regular pulse, and he did…I was greatly struck by this fact, and afterward tried this sort of treatment on many cases of auricular fibrillation. My success was disappointing, in that quinine abolished auricular fibrillation in only a few cases, and these cases only when the onset of this form was quite recent, never when it was of several years' duration” (Moe, 1970)*.

Frey in 1918 reported that the d-isomer of quinine, quinidine, also found in cinchona bark, exerts more powerful effect against AF than the l-isomer quinine (Moe, 1970). The empiricism in AAD therapy continued through the thirties by the collaborating team of anesthesiologists and cardiac surgeons during open heart surgery. They discovered in 1936 that the application of the local anesthetic agent, procaine, either at the epicardial surface of the heart or by intravenous injections in patients during cardiac surgery provided “protection” against VT/VF by reducing “cardiac irritability” (Mautz, 1936). This success led to the ultimate development of the longer and more potent acting analog of procaine, procainamide with lesser central nervous system toxicity. Encouraged by the results of the local anesthetic procaine, intravenous lidocaine was introduced by Hitchcock and associates for the rapid conversion of VT to sinus rhythm in the late fifties (Hitchcock and Keown, 1959). An early attempt to a “mechanistic” approach to combat VT/VF was reported by Harris and Kokernot (1950). These authors suggested the presence of a similarity between the ectopic rapid ventricular discharges in dogs after coronary artery occlusion and the focal epileptogenic discharges in the brain of patients with epilepsy:

*“…Drugs that have proved effective in preventing focal seizures might suppress ectopic ventricular discharges which accompany acute myocardial infarction” (Harris and Kokernot, 1950)*.

Using dilantin (diphenylhydantoin, or DPH), a drug with demonstrated efficacy against epileptic seizures in humans, these authors recorded a measure of success in dogs with acute ischemic VT/VF by asserting that: “*The excitability and automaticity of cardiac tissues are markedly similar to the changes produced in excitability and automaticity of nerve and skeletal muscle”* (Harris and Kokernot, 1950). However, others have suggested that DPH's direct actions on cardiac Purkinje fibers (shortening of the APD, reducing spontaneous phase 4 depolarization and firing rate) “*were sufficient to suggest an explanation for it anti-arrhythmic effect on ventricular arrhythmias in vivo”* (Bigger et al., 1968). It is possible that both central and direct actions of DPH may conspire to bring about a measure of antiarrhythmic effect (Karagueuzian, 1995). Currently DPH has very limited use against VT/VF (Bäckman et al., 1989).

## AAD class actions: from Huggins to Singh-Vaughan Williams to Harrison to Sicilian Gambit and back

The first classification of AADs was made in 1949 by Huggins and associates (Huggins et al., 1949). These authors grouped the drugs into three categories based on the drugs'(1) local anesthetic effect; (2) adrenolytic potency and (3) coronary artery relaxing effects (Huggins et al., 1949). This first ever AAD classification, surprisingly not acknowledged in any of the subsequent AAD classifications, was the origin of all subsequent classifications. These pioneering authors tested the efficacy of what they called “three groups” of drugs against epinephrine-induced ventricular fibrillation (VF) in chloroform anesthetized dogs (Huggins et al., 1949). The “first group of agents” included the coronary vasodilators (i.e., sodium nitrite, aminophylline, papaverine, and quinacrine); “the second group” included drugs that decreased myocardial excitability (local anesthetic action) such as procaine, quinidine sulfate; and the “third group of agents” manifested sympatholytic activity (i.e., beta blocking effect) such as priscol and dibenamine (Huggins et al., 1949). When the vasodilator group was found ineffective it was dropped from the list leaving (1) the myocardial excitability depressants and (2) the beta adrenergic receptor blockers.

With the introduction in the late 1940s of cellular transmembrane action potential recordings with the glass microelectrode technique from atrium and ventricle, Singh and Vaughan Williams introduced a “third class” of AAD action in 1970 when they discovered that d-sotalol's antiarrhythmic efficacy was independent of beta blocking effect. Instead they attributed d-sotalol's antiarrhythmic efficacy to the drug's ability to prolong cardiac cellular action potential duration (APD), thus proposing a “third class” of AAD action: prolongation of the APD (Singh and Vaughan Williams, 1970a). This proposal was based on two additional observations. First, on shortened APD during atrial fibrillation (AF) initiated by thyrotoxicosis and resolution of the AF with thyroidectomy associated with prolonged APD (Vaughan Williams, 1970). Second, based on the effects of amiodarone, initially introduced as an antianginal agent (Charlier et al., 1968) and later as an AAD (Charlier et al., 1969) by prolonging atrial and ventricular APD (Singh and Vaughan Williams, 1970a,b). The similarities of d-sotalol and amiodarone to thyroidectomy in suppressing arrhythmias by prolonging the APD led these authors to conclude that the “*prolongation of the APD may be considered as a third type of action reducing the probability of the arrhythmia”* (Vaughan Williams, 1970). However, an apparent dilemma was created when it was discovered that both quinidine and procainamide, two Class I myocardial depressant drugs, also prolong the APD similar to Class III drugs like d-sotalol and amiodarone. These authors however, asserted that the myocardial depressant effect of excitability caused by Class I AADs emerges *prior* to the APD prolongation, therefore attributing quinidine's antiarrhythmic efficacy solely to its myocardial depressant effect of excitability rather than to its APD prolonging effect (Szekeres and Williams, 1962; Singh and Vaughan Williams, 1970a). However, the later confirmation of significant directional differences of the effects of Class I AADs on cellular APD necessitated their separations into three “sub-classes” on the basis of their ability to prolong (Class 1A action), shorten (Class 1B action), or have no effect (Class 1C action) on the APD (Harrison, 1985; Table 1).

**Table 1:** **Updated classification of antiarrhythmic drugs**.

Class I		
IA	Peak I_Na+_ current blockers	Quinidine, Procainamide, Disopyramide, Cibenzoline
IB		Lidocaine, Tocainide, Mexiletine, Ethmozine, Ranolazine^*^
IC		Encainide, Lorcainide, Flecainide, Propafenone
Class II	Beta blockers	Propranolol, Esmolol, Timolol, Atenolol, Metoprolol, Carvedilol
Class III	K^+^ channel blockers	Amiodarone, d-Sotalol, Ibutilide, Dofetilide, Dronaderone
Class IV	Ca^2+^ channel blocker	Verapamil, Nifedipine, Diltiazem, Bepridil
Class V	I_f_ blocker, and other agents	Ivabradine, Adenosine, Digitalis, Magnesium Sulfate
Class VI	Late I_Na_ and late I_Ca,L_ blockers	GS-458967 Roscovitine

* *Given the structural similarity to lidocaine, ranolazine was initially grouped as Class 1B. However, depending on the relative contributions of ranolazine's block of IKr vs. INa-L the APD may prolong (Class 1A), shorten (Class IB) or remain unchanged (Class 1C)*.

There are great deals of overlaps between drugs of different classes (Charlier, 1970) making rigid categorizations of the current AADs a difficult task. Moreover, the classification is based on the effects of AADs on cardiac action potential characteristics rather than on clinical arrhythmias. For example, Class 1C drug effect indicates “reduction of peak Na current (I_Na_) and no change in APD” and says nothing about its therapeutic value. In short, the classification provides a useful conversational shorthand with no distinct clinical value. In fact, the shortcomings of the AAD classification was recognized back in 1970 by Singh and Vaughan Williams in their original proposal of Class III AAD action:

*“The effect of anti-arrhythmic agents on various parameters of cardiac function in vitro and in vivo have made it possible to classify the drugs into groups which do or do not have certain clearly definable pharmacological actions though these actions do not necessarily determine anti-arrhythmic effectiveness” (Singh and Vaughan Williams, 1970a)*.

*Class IV AADs* The discovery of verapamil in the seventies, a Ca channel blocker (CCB) (Kohlhardt et al., 1972), that exerts potent AA efficacy against diverse experimental models of arrhythmias including myocardial ischemia, A–V nodal reentrant tachycardia and digitalis toxicity necessitated the introduction of CCB as a fourth class (Class IV) of AAD action (Kohlhardt et al., 1972; Rosen et al., 1975; Table 1), replacing the “centrally acting” Class IV AADs (i.e., Dilantin) originally proposed by Vaughan Williams (1970). Unfortunately, the concentrations of Class IV AADs that suppress arrhythmias cause severe negative inotropic effects that greatly diminish their therapeutic value, particularly in patients with compromised cardiac function (Rosen et al., 1975).

### Class V AADs

Class V agents include molecules with diverse pharmacological actions, including digitalis, adenosine, ivabradine, and magnesium chloride. Some agents in this Class like vernakalant, which blocks peak Na current and is effective against AF (European Heart Rhythm et al., 2010), can be placed in several classes, while others like ivabradine (sinus node pacemaker I_*f*_ blocker), cannot be listed under any of the four classes (Thireau et al., 2011). Same goes for digitalis that increases vagal activity via its action on the central nervous system, thus decreasing the conduction of electrical impulses through the AV node (van Veldhuisen et al., 1996). As for adenosine, its actions are, for the most part, due to the direct activation of the G protein-gated inward rectifying potassium channels and antagonist of cAMP-stimulated ion currents such as the pacemaker current and the L type calcium current (I_Ca,L_) (Lerman and Belardinelli, 1991; Shryock and Belardinelli, 1997). The cardiac actions of adenosine include slowing of sinus rate, depression of atrioventricular (AV) nodal conduction, suppressing AV nodal reentrant tachycardia (Belhassen et al., 1988; Camm and Garratt, 1994; Prystowsky et al., 2015). Finally, MgCl_2_ is found effective against EAD-mediated triggered activity in canine Purkinje fibers (Bailie et al., 1988) and is suggested as a potential adjunctive therapy for cardiac arrhythmias in humans (Baker, 2016).

## The Sicilian Gambit “classification”

The dissatisfaction with the current AAD classification systems and the disappointing results with two major clinical trials, the Cardiac Arrhythmia Suppression Trial (CAST) testing the efficacy of Class I AADs (Echt et al., 1991) and the Survival With Oral D-Sotalol (SWORD) clinical trial testing the efficacy of Class III drug (Waldo et al., 1995) led a group of established cardiac electrophysiologists to propose supplementing the current AAD class actions by incorporating the broad pharmacodynamic actions of each AAD (Task force of the working group on arrhythmias of the European Society on C, 1991). While informative, this new approach, known as the Sicilian Gambit, was argued to be “*essentially similar”* to the original classification (Vaughan Williams, 1992). Indeed, and two decades later members of the Group of the Sicilian Gambit Investigators, had this to say:

*“The Sicilian Gambit…was not meant in aggregating drugs into categories, instead it was intended to provide background information so to challenge thought and investigation rather than to resolve issues” (Rosen and Janse, 2010)*.

The AAD therapy remains largely empirical. The link between specific ionic effect(s) of a drug and suppression of a specific arrhythmia mechanism(s) remain(s) incompletely understood (Weiss et al., 2015). All AADs block with differing degrees of potencies, multiple ionic currents that prevent precise understanding as to which specific drug-induced ionic change(s) in the heart is or are responsible for the outcome. The lack of mechanistic understanding of the success or failure with AAD therapy, greatly diminished the enthusiasm in innovative AAD research. At the present there are important gaps in our understanding of the ionic mechanism(s) of the arrhythmia sought for therapy. As articulated by van Hamel:

*“Manifestations of an arrhythmogenic substrate and its triggers can be almost completely suppressed for a while -in clinical terms defined as a therapeutic success and therefore satisfying- without understanding in detail its mechanisms, anatomical extent, natural course and potential complications. This attitude toward arrhythmia management offers only short-term solutions and still worse, suppresses our intellectual curiosity” (van Hamel, 2003)*.

Indeed, it is clear that better understanding of the molecular pathological mechanisms of the arrhythmia would result in a better and more effective management of the arrhythmia because of possibility of specific drug targeting of the pathological mechanisms of the arrhythmia. Unfortunately, at the present there remain important gaps concerning the comprehension of these mechanisms preventing a rational and specifically targeted drug therapy of these arrhythmias.

**Figure 1:**
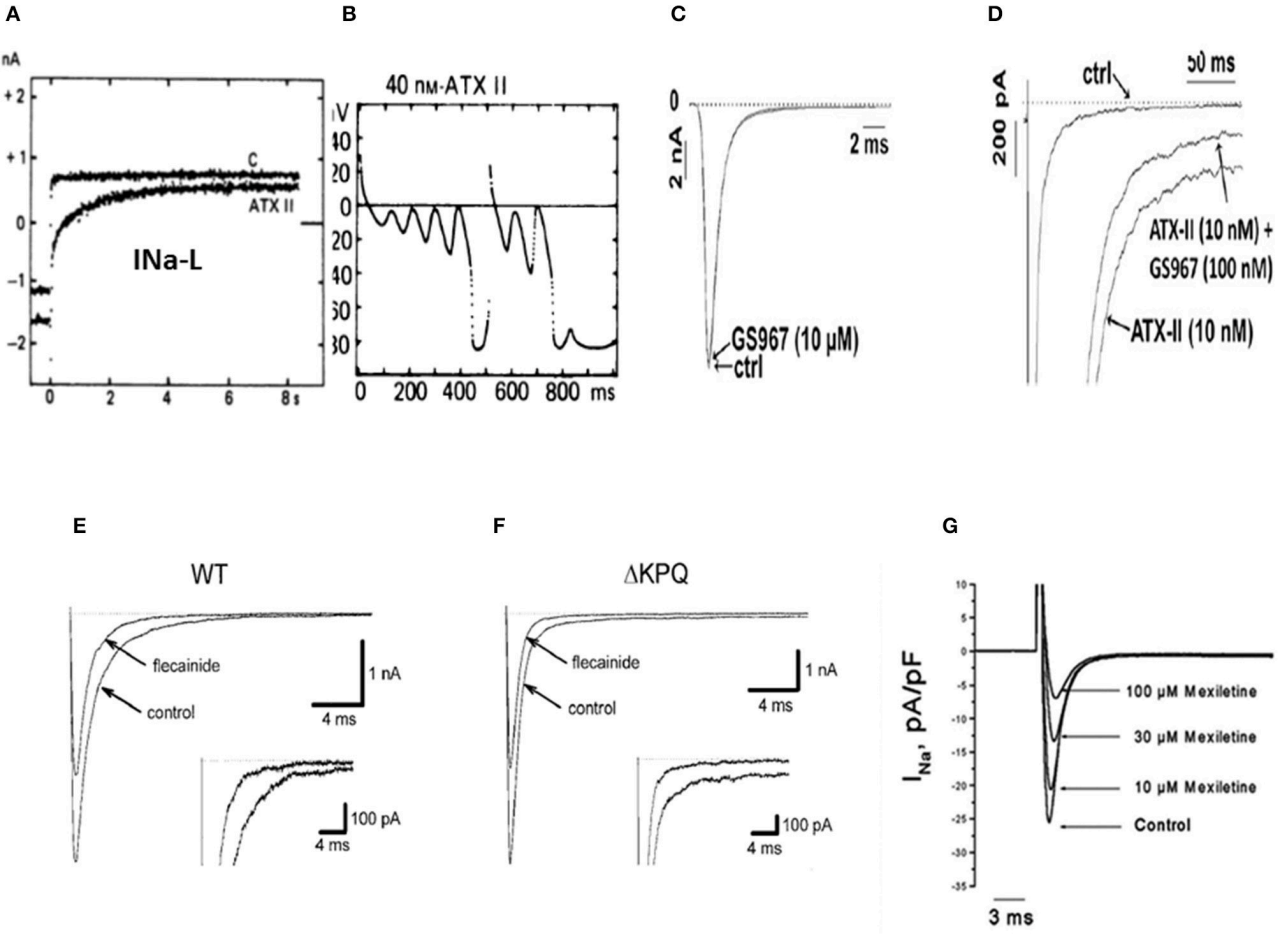
**Enhanced I_**Na-L**_, initiation of EADs and their selective suppression with specific I_**Na-L**_ blockers (A)** shows increased I_Na-L_ induced with ATXII in isolated guinea-pig ventricular myocyte initiating EADs and triggered activity **(B)** (From Isenberg and Ravens, 1984). **(C)** Shows absence of effect of the selective I_Na-L_ blocker, GS-967 on peak I_Na_ induced with ATXII in isolated rabbit ventricular myocyte while selectively inhibiting the I_Na-L_ **(D)**. (From Belardinelli et al., 2013) **(E)** Shows 50% reduction of peak I_Na_ in WT and **(F)** in LQT3 mutant Na channel (ΔKPQ) in HEK cells with sub-therapeutic (0.1 μM) concentrations of flecainide while also causing non-specific reduction in the I_Na−L_, (insets) (From Nagatomo et al., 2000). **(G)** Shows mexiletine's concentration-dependent reduction of the peak I_Na_ in isolated rabbit ventricular myocytes with no specific inhibitory effect on the I_Na-L_ (From Gao et al., 2013).

**Figure 2:**
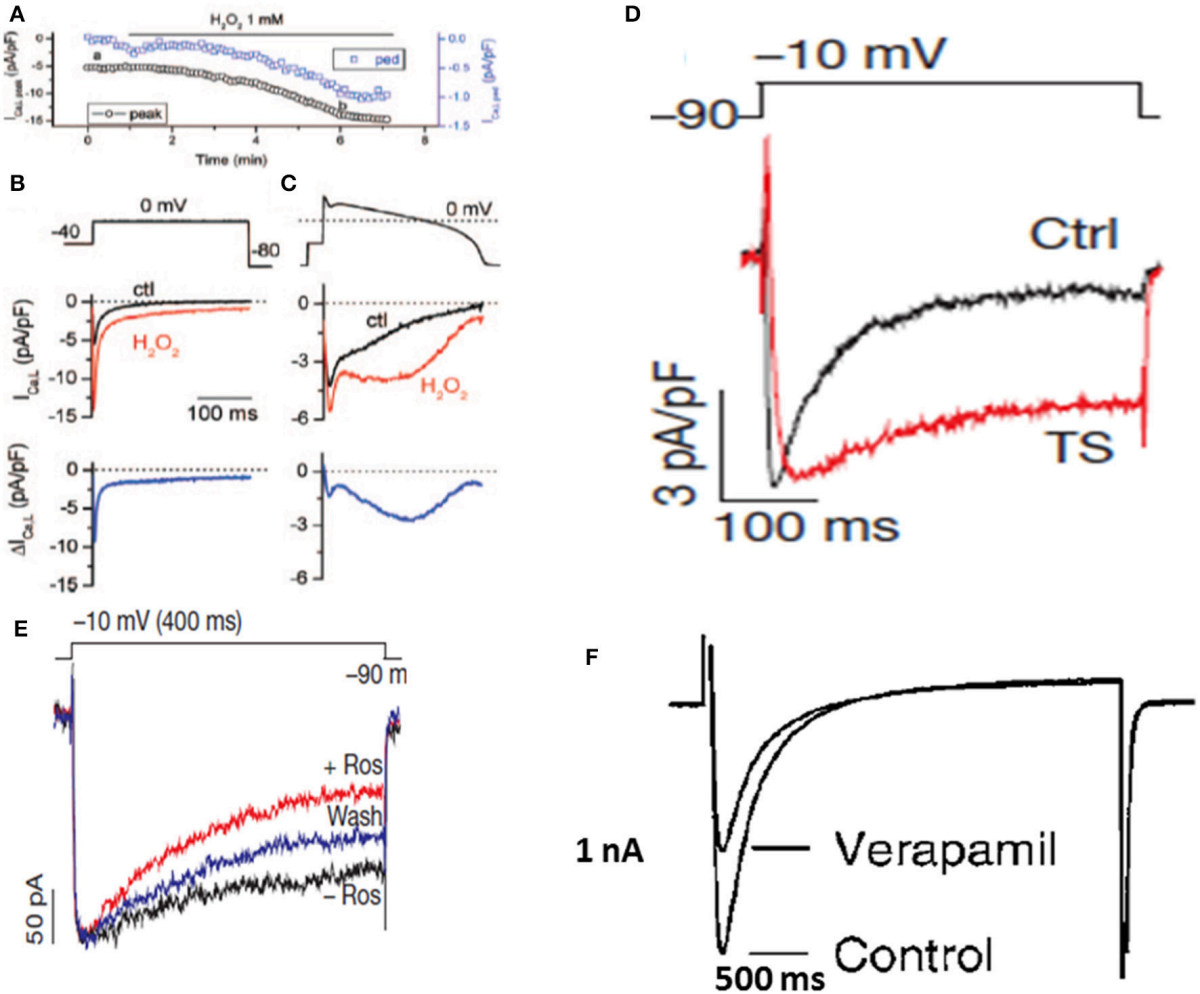
*****Enhanced late I***_***Ca,L***_***and its selective block with roscovitine but not with verapamil***. (A–C)** show the time course of the peak and the pedestal (ped) I_Ca,L_ during a 300-ms voltage clamp pulse to 0 mV (voltage protocol shown in B). **(B)**. Voltage clamp pulse (above) and superimposed current traces showing I_Ca,L_ before (black) and ~5 min after perfusion of 1 mmol/L H_2_O_2_ (red). The difference current is shown in the bottom trace. **(C)**, Same as in **(B)** but with an AP clamp waveform replacing the square voltage clamp pulse (From Xie et al., 2009). **(D)** Shows an iPSC-derived cardiomyocyte from a patient with TS showing increased late I_Ca,L_ (From Yazawa et al., 2011). **(E)** Shows selective block of the late I_Ca,L_ with roscovitine in a cardiomyocyte derived form a TS patient without affecting the peak I_Ca,L_ (From Yazawa et al., 2011). **(F)** Shows lack of selective block of the late I_Ca,L_ with verapamil while causing severe depression of the peak I_Ca,L_ (From Nawrath et al., 1998).

The recent recognition of the pathological increases in the late Na current (I_Na-L_) and I_Ca,L_ as “vulnerable parameters” for early afterdepolarizations (EADs)-mediated VT/VF provides an opportunity to specifically target the dynamical arrhythmia mechanism. Importantly, the suppression of these VT/VFs with specific prototype blockers of I_Na-L_ and the late I_Ca,L_ without affecting their peaks, provide a rational and effective AAD therapy that specifically target the arrhythmia mechanism. Under normal conditions, voltage-gated sodium channels (NaV1.5) open suddenly upon depolarization permitting entry of Na^+^ that peaks in ≈1 ms (I_Na_) then rapidly decays to baseline as most Na^+^ channels quickly close (inactivate) during the plateau phase of the action potential (AP). While a very small persistent I_Na_ current may be present under normal conditions, however, under diseased conditions I_Na_ inactivation can be greatly slowed, independent of its peak, providing persistent late Na current during the plateau phase of the AP three to tenfold over the level of normal conditions (Belardinelli et al., 2015). It is difficult to have a precise idea in absolute terms the level at which I_Na-L_ increases as this level is profoundly affected by diverse factors including differences in the clamp protocol, species use, age (neonatal vs. adult), temperature (room vs. body temperature), nature of the stress (i.e., heart failure, ATX, hydrogen peroxide, toxins like aconitine). Importantly the level of the I_Na-L_ depends on the timing when the “late” I_Na_ is measured, 50 or 100 ms after the clamp pulse or during the steady state (Ward and Giles, 1997; Song et al., 2006; Maltsev and Undrovinas, 2008; Undrovinas and Maltsev, 2008; Antzelevitch et al., 2014; Belardinelli et al., 2015; Wagner et al., 2015). It is estimated that during the steady-state pathological increases of the I_Na-L_ can reach up to 20-fold (roughly about 100 pA) of control as shown in Figure 1D. Similarly as in the case of the I_Na_, the I_Ca,L_ inactivation rate can also be considerably slowed under diverse cardiac conditions secondary to CaMKII activation generating an enhanced late inward depolarizing current. This I_Ca.L_ component, particularly relevant during the plateau and the repolarization phases of the cardiac AP, became known as the late I_Ca,L_ (Madhvani et al., 2015; Markandeya and Kamp, 2015). Under normal conditions late I_Ca,L_ is minimal however, under pathological conditions its amplitude increases significantly. As in the case of the I_Na-L_ the absolute values of the late I_Ca-L_ varies greatly due to the same factors mentioned for I_Na-L_ plus the rundown phenomenon that is associated with the LCCs (Yue et al., 1990; Xie et al., 2009; Madhvani et al., 2011, 2015; Kim et al., 2016). Figures 2A,B shows variations in the absolute amplitude of the late I_Ca-L_ depending on the conditions of the study. Diverse acquired and congenital cardiac conditions at risk of developing VT/VF including heart failure, myocardial infarction and cardiomyopathies manifest pathological rises in these late inward currents, often exacerbated by activation of CaMKII signaling (Maltsev et al., 2007; Mollova et al., 2015). In the ensuing sections, animal and human models of VT/VF in which CaMKII activation plays a key role are presented. Electrophysiological studies scaling from isolated myocytes to intact hearts show that downstream increases in the I_Na-L_ and late I_Ca,L_ play key roles in CaMKII-driven VT/VF providing a rational and effective AAD class action not considered in previous AAD classifications.

## Enhancement of I_Na−L_ and late I_Ca,L_ via CaMKII signaling

Evidence accumulated over the last two decades has provided novel mechanistic insights into the ionic mechanisms of triggered VT/VF that can be specifically targeted by small drug molecules. The multifunctional and ubiquitous enzyme CaMKII is a well demonstrated sensor of calcium and redox signaling that enhances the I_Na-L_ and late I_Ca,L_ and plays a key role in the pathogenesis of heart failure and arrhythmias both in humans and in animal disease models (Erickson et al., 2008; Xie et al., 2009; Belardinelli et al., 2015; Hund and Mohler, 2015; Pezhouman et al., 2015; Mustroph et al., 2016). Patients with heart failure, hypertrophy and cardiomyopathies manifest sustained hyperactivity of CaMKII (Erickson et al., 2008, 2011; Mollova et al., 2015) promoting VT/VF (Swaminathan et al., 2012; Luczak and Anderson, 2014). Isolated myocytes studies have shown that CaMKII activation enhances the I_Na-L_ and the late I_Ca,L_(Xie et al., 2009; Belardinelli et al., 2015) promoting EADs and triggered arrhythmias (Morotti et al., 2014; Foteinou et al., 2015; Pezhouman et al., 2015). Consistent with these findings, transgenic CaMKII overexpression in the mouse leads to development of heart failure, while CaMKII deletion prevents onset of heart failure following transaortic constriction (Maier et al., 2003; Zhang et al., 2003; Backs et al., 2009; Ling et al., 2009). CaMKII-based therapy of cardiac arrhythmias however, is inherently difficult due to its vast signaling network requiring a careful balance between the therapeutic benefit and the potential off-target effects (e.g., neuronal or metabolic; Hund and Mohler, 2015). An alternative would be specific down-stream targets of the CaMKII pathway known to play key role in promoting arrhythmias. The I_Na-L_ and the late I_Ca,L_ are two demonstrated down-stream targets that play a key role in EAD-mediated arrhythmias (Morita et al., 2011a; Madhvani et al., 2015). Indeed, dynamic-clamp and simulation studies have shown that selective enhancement of the late I_Ca,L_ or the I_Na-L_ without changing any other ionic currents is sufficient to promote EADs and triggered activity (Madhvani et al., 2011, 2015). These findings indicate that the other downstream effects of CaMKII activation including, phosphorylation of ryanodine receptor, (RyR2), phospholamban (PLB), sarco/plasmic-endoplasmic Ca-activated ATPase type 2a (SERCA2a), and increased Na-Ca exchanger current (I_NCX_) (Marx et al., 2000; Hund and Mohler, 2015; Mustroph et al., 2016), while potentially modulating the genesis of EADs do not appear necessary for oxidative EAD formation in isolated ventricular myocytes (Madhvani et al., 2011, 2015) and EAD-mediated VT/VF in intact hearts (Morita et al., 2011a; Pezhouman et al., 2016). The Ca handling proteins however, could play an important role both in atrial and ventricular tissue in promoting DAD-mediated fibrillation as shown in human and animal studies (Voigt et al., 2012; Luczak and Anderson, 2014).

### The late Na current (I_Na−L_)

The pathological rise of the late Na current known as late I_Na_ (I_Na-L_), emerges under cardiac conditions associated with increased risk of developing VT/VF(Belardinelli et al., 2015) secondary to the activation of CaMKII signaling pathway (Erickson et al., 2011) and mutations of Nav1.5 channels (Bennett et al., 1995; Ulbricht, 2005). The cardiac conditions associated with increased risk of VT/VF include human congenital LQT3 (Bennett et al., 1995; Ulbricht, 2005) and laminopathy (Markandeya et al., 2016) and a host of acquired chronic cardiac diseases including heart failure (Maltsev et al., 2007), myocardial ischemia (Belardinelli et al., 2013), increased pro-oxidant states (Ward and Giles, 1997; Song et al., 2006; Xie et al., 2009), and hypertrophic cardiomyopathy (Coppini et al., 2013; Belardinelli et al., 2015). It however must be emphasized that in the congenital LQT3, the I_Na-L_ is not necessarily CaMKII-dependent. The importance of pathological rise of the I_Na-L_ in the genesis of EADs and EAD-mediated arrhythmia has been systematically studied in isolated cardiac myocytes using potent toxins like sea anemone toxin II (ATX-II) that specifically enhance the I_Na-L_ with minimal effect on other ionic currents (Isenberg and Ravens, 1984; Belardinelli et al., 2015; Figures 1A,B). The key role played by the selective increase of the I_Na-L_ current in EAD-mediated arrhythmias is demonstrated using the highly selective and potent (IC50 of 134 nM) blocker of the I_Na-L_, GS-458967 (GS-967) (Belardinelli et al., 2013) in suppressing I_Na-L_ as shown in Figures 1C,D). This selective effect of GS-967 on I_Na-L_ is fundamentally different from the traditional Class 1 AADs like flecainide and mexiletine that potently block the peak I_Na_ as shown in Figures 1E–G. The demonstrated efficacy of GS-967 to suppress EAD-mediated arrhythmias provided a direct link between a specific molecular action of a drug (i.e., “gating modifier” specific block of I_Na-L_) and a dynamical arrhythmia mechanism (enhanced I_Na-L_-mediated EADs) (Qu et al., 2013). The term “gating modifier” has been used to refer to the mode of action of toxins that alter the voltage dependence of voltage gated ion channels by binding to structures that undergo conformational changes during gating, e.g., the voltage sensing domains (Catterall et al., 2007; Kalia et al., 2015). Here, we used the term gating modifier to refer to drug action that alter channel opening and channel closing without directly blocking the pore. Modulation of the gating of brain sodium channels accounts, at least in part, for the ability of several anti-epileptic drugs including lamotrigine to suppress epileptic attacks. Interestingly it is shown that lamotrigine exerts its effect by binding to the two voltage sensors, segments S4, of the domains III and IV of Nav channel that potently suppress EAD-mediated triggered activity (epileptic discharges) without affecting ordinary nerve action potential firing (Rogawski and Loscher, 2004). This finding suggest that the voltage sensor domain of the voltage gated Na channels can be a drugable target for antiepileptic drugs. Indeed, in a transgenic mouse model of epilepsy (*Scn2a*^Q54^), the I_Na-L_ was found to be increased significantly from 1 to 3 percent of peak I_Na_ and importantly the specific I_Na-L_ blocker prototype GS-967, mitigated epileptic attacks (Anderson et al., 2014). These findings suggest that the gating modifiers of Nav channels may be effective and target specific anti-epileptics (Anderson et al., 2014; George, 2015).

Unfortunately, the current Classes of AADs are incapable to selectively discriminate between the pathological I_Na-L_ and the normal peak I_Na_ necessary for normal AP upstroke velocity as they equally suppress both the early and late components of the I_Na_. For example, flecainide (Class 1C AAD) blocks both the peak I_Na_ and I_Na-L_ in wild type and in LQT3 mutant Na channels as shown in Figures 1E,F. Similarly, mexiletine, a Class 1B AAD has similar effects as flecainide on peak and I_Na-L_ (Figure 1G; Gao et al., 2013). The partially selective I_Na-L_ blocker ranolazine, while suppressing the I_Na-L_ and EAD-mediated VT/VF (Sicouri et al., 2008; Morita et al., 2011a) was tentatively suggested to fit Class 1B, due to its structural similarity to lidocaine (Thireau et al., 2011). However, ranolazine blockade of I_Kr_ which tends to prolong the APD and the QT interval may well place it in the Class IA category, like quinidine (Table 1).

### The late L-type Ca current (I_Ca,L_)

Cardiac conditions that increase the late I_Ca,L_ are invariably associated with increased risk of VT/VF, including heart failure (Mewes and Ravens, 1994; Cooper et al., 2010), congenital LQT8 mutations and Timothy Syndrome (TS) (Figure 2A; Limpitikul et al., 2014; Marsman et al., 2014; Dick et al., 2016) increased pro-oxidant states, (Figure 2B; Xie et al., 2009) aging, (Zhou et al., 1998; Salameh et al., 2010), and Duchenne muscular dystrophy (Koenig et al., 2011; Viola et al., 2013) and in stem cell derived cardiomyocytes from patients with Timothy syndrome (Yazawa et al., 2011; Figure 2D).

**Figure 3:**
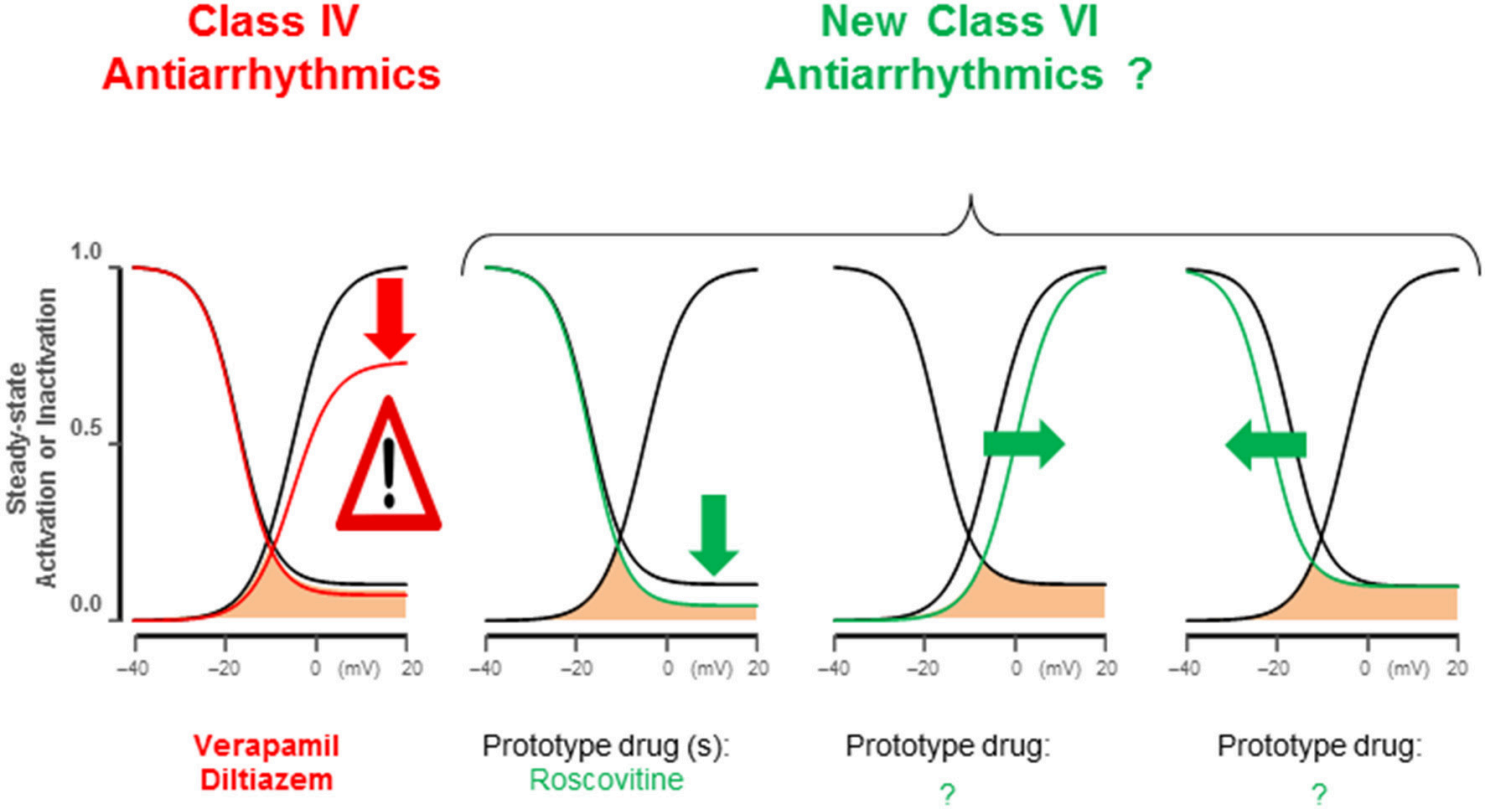
**Late I_**Ca,L**_ antiarrhythmic modifications**. Class IV antiarrhythmics block the L-type channels indiscriminately reducing peak and late I_Ca,L_, suppressing EC-coupling. I_Ca,L_ gating modifications identified by dynamic clamp studies (Madhvani et al., 2011, 2015) specifically reduce late I_Ca,L_, potently abolishing EADs without affecting maximum Cav1.2 open probability; therefore peak I_Ca,L_ remains largely preserved. These maneuvers include: (1) pedestal reduction from 10 to 4%; (2) a 5 mV depolarizing shift of steady-state activation; (3) a 5 mV hyperpolarizing shift of SS-inactivation. Roscovitine enantiomers can reduce late I_Ca,L_ (pedestal).

Electrophysiological studies in isolated cardiac myocytes have shown that an increase in the late I_Ca,L_ causes EADs (Xie et al., 2009; Figure 2C). Most EADs develop between −40 and 0 mV, a range of membrane potentials commonly referred to as the window current region, where the steady-state activation and steady-state inactivation curves overlap. Within the I_Ca,L_ window region, a fraction of Ca channels are not fully inactivated maintaining a non-zero open probability responsible for the late Ca current. Recent studies based on dynamic clamp data provide compelling evidence that interventions reducing late I_Ca,L_ by either by (1) shifting the I_Ca,L_ steady-state activation in the depolarizing direction by <5mV, (2) shifting the steady-state inactivation curve in the hyperpolarizing direction by (<5 mV), or (3) reducing the non-inactivating (pedestal) component (Madhvani et al., 2015; Figure 3) potently suppresses EADs and therefore carry a strong therapeutic value for suppressing EAD-mediated VT/VF. Traditional CCBs like verapamil or nifedipine can suppress oxidative EADs by blocking I_Ca,L_ (Xie et al., 2009) however, their suppressant effect of EADs comes at a cost of considerable reduction of the peak I_Ca,L_ as shown in Figure 2E. The simultaneous block of peak and late I_Ca,L_ by a drug greatly diminishes the therapeutic value particularly in patients with compromised cardiac function (Figure 2F; Nawrath et al., 1998). Importantly, the dynamic clamp studies of Madhvani et al. (2015) provide compelling evidence that the blockade of peak I_Ca,L_ is not only undesirable but also not necessary for EADs suppression! These studies made it clear that the selective reduction of the late I_Ca,L_ can preserve Ca transient and normal EC coupling, while at the same time potently suppressing the EADs. These findings suggest that a new class of drug action, “gating modifiers,” as in the case of I_Na-L_, that induce subtle changes of the gating properties of the LTCC and Nav channels without blocking their pores suppress EAD-mediated VT/VF without adversely affecting contractility. Interestingly, the identification roscovitine, a purine derivative (anticancer agent) able to selectively reduce the late I_Ca,L_ without affecting the peak I_Ca,L_ represents a turning point in the electropharmacology of AADs (Yarotskyy and Elmslie, 2007; Yazawa and Dolmetsch, 2013; Figure 2D).

### Intact heart models of EAD-mediated VT/VF via CaMKII signaling

**Figure 4:**
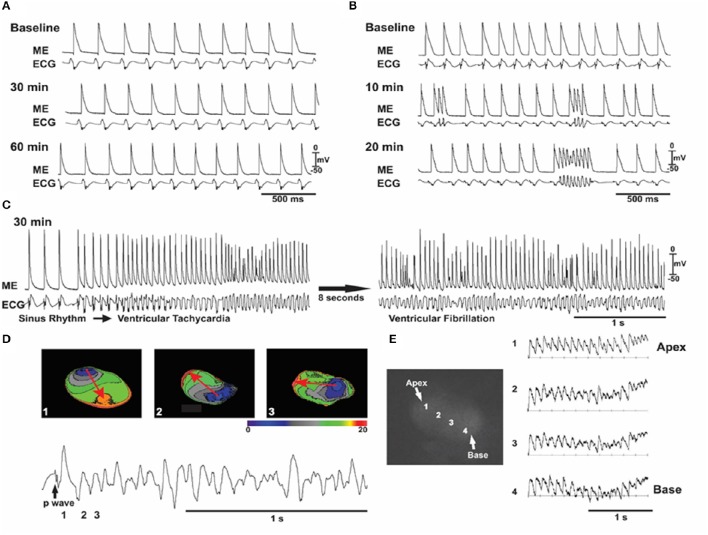
**Oxidative stress with angiotensin II (ANG II) induces early afterdepolarizations (EADs), VT/VF in an aged but not young rat hearts**. In all **(A–C)**, simultaneous microelectrode (ME) and pseudo-ECG recordings in a young heart **(A)** and an aged heart **(B,C)** exposed to ANG II (2 mol/l). A: representative experiment in a young heart showing that sinus rhythm persists throughout 60 min of ANG II perfusion. **(B,C)** Recordings from an aged heart showing the emergence of EADs, triggered activity, and VF over the indicated time course. **(D)** Three isochronal activation maps, with the first being the last sinus beat (beat 1) before the onset of the VF shown at the bottom and indicated as 1 in the ECG (bottom). The sinus beat was followed by two consecutive focal activations that arose from the base of the heart (beats 2 and 3). The red arrows in **(D)** indicate the direction of propagation. The focal activation lasted for 8–10 beats (the initial VT phase) and then degenerated to VF, as shown in **(E)** with four optical action potentials (APs; labeled 1–4) shown in the adjacent schema (From Bapat et al., 2012).

We and others have shown that oxidative activation of CaMKII by hydrogen peroxide (H_2_O_2_) increases the I_Na-L_ and late I_Ca,L_ and readily promotes EADs and triggered activity in isolated rat and rabbit ventricular myocytes (Ward and Giles, 1997; Song et al., 2006; Xie et al., 2009; Madhvani et al., 2015). At tissue level however, EADs may be suppressed by source-sink mismatches arising from cell-to-cell coupling. That is, the small inward current which is sufficient to reverse repolarization and cause an EAD at the isolated myocyte level will be “diluted” into adjacent repolarizing myocytes at the tissue level (unless they are also simultaneously primed for an EAD), thereby preventing EAD formation. The isolated single cell vs. tissue level vulnerability to EAD formation was investigated by examining the arrhythmogenic effects of hydrogen peroxide (H_2_O_2_) in Langendorff rat and rabbit hearts. Oxidative stress was shown to readily induce EADs at the isolated myocyte level in these two species. Consistent with the predicted suppressive effects of well-coupled tissue on EADs, we found that oxidative stress with H_2_O_2_ failed to induce any ventricular arrhythmias in normal well-coupled young/adult (~4 months old) rat and rabbit hearts (Figure 4A). Even a tenfold increase in the level of peroxide failed to promote EADs in these normal hearts (Morita et al., 2009). However, aged, 24–26 months old rat hearts, manifest considerable heterogeneous increase in interstitial tissue fibrosis (10–90%), with reduced cell-to-cell gap junctional couplings via connexin43 (Cx43), H_2_O_2_ exposure consistently promoted EADs and triggered activity leading in >90% of the aged fibrotic hearts to VT/VF. (Figures 4B–E) Similarly, middle-aged, 3–5 years old, (normal life span 8–12 years) rabbit hearts, with lesser rise in fibrosis (5–35%) than in aged rat hearts and developed lesser incidence (~50%) of EAD-mediated triggered VF (Morita et al., 2009; Sato et al., 2009). Although other aging-related factors may play a role in EAD-mediated VF, we hypothesized that reduced cell-to-cell gap junctional coupling in fibrotic aged hearts promotes oxidative EAD-related arrhythmias by favorably altering source-sink relationships. Indeed, computer simulation in 1, 2, and 3D cardiac tissue incorporating fibrosis fully supported this interpretation (Xie et al., 2010; Morita et al., 2011a). The involvement of peroxide, as a redox sensor of CaMKII, is validated by demonstrating similar VT/VF outcomes with more proximal oxidative signaling using acute angiotensin II perfusion (Bapat et al., 2012) or by mimicking distal consequences of oxidative stress, such as glycolytic inhibition (Corretti et al., 1991), all promoting EAD-mediated VT/VF as in the case of H_2_O_2_ (Morita et al., 2011b). Interestingly both oxidative stress with peroxide (Pezhouman et al., 2014a) or downstream oxidative injury with glycolytic inhibition (Ono et al., 2007) in aged atria promote atrial EADs, tachycardia and fibrillation (AT/AF) in aged rat atria as in aged rat ventricles. However, it remains to be seen if block of the late inward currents would also prevent and suppress AT/AF in other species such as rabbit, goat, sheep and dog models of AT/AF.

## Enhanced late inward currents promote both focal triggered and reentrant arrhythmias

**Figure 5:**
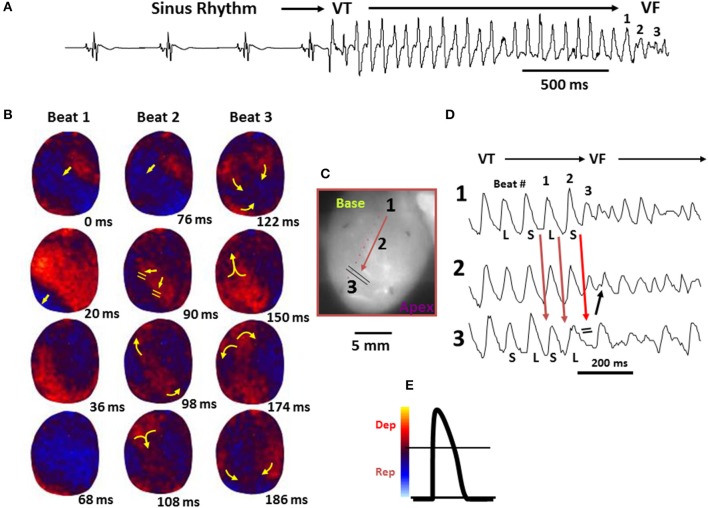
**Spontaneous initiation of ventricular tachycardia (VT)/VF in an aged rat heart exposed to 0.1 mM H_**2**_O_**2**_. (A)** ECG showing the last 5 sinus beats before the sudden onset of VT leading to VF. **(B)** voltage snapshots of the last beat of the VT (beat 1) and of the first 2 beats of the VF (beats 2 and 3). In each snapshot, activation time (in ms) is shown at the bottom right with time 0 (arbitrary) coinciding with the onset of beat 1. The red color in the snapshots represents depolarization (Dep) and the blue repolarization (Rep) as shown in **(E)**. The yellow arrows in the snapshots represent the direction of the wavefront propagation with double horizontal lines denoting the site of conduction block. The VT originates from a focal site at the LV base and propagates as single wavefront toward the apex and undergoes functional conduction block at site 3. The two lateral edges of the front, however, continue to propagate laterally (snapshot, 98 ms) forming figure-eight reentry (snapshot, 108 ms). During the second reentrant wavefront, another wavefront emerges from the apical site of the LV (snapshot, 122 ms), disrupting the activation pattern and signaling the onset of VF. **(D)** 3 optical action potentials (APs; labeled 1, 2, and 3) recorded from sites identified on the heart silhouette **(C)**. The 2 downward-pointing blue arrows indicate the direction of propagation from site 1 to site 3 with the red downward-pointing arrow showing block at site 3, followed by retrograde activation (upward-pointing arrow). Notice the emergence of spatially discordant AP duration (APD) alternans preceding conduction block at site 3 when the front with short APD (S) at site 1 encroaches a site (site 3) with long APD (L) (From Morita et al., 2009).

**Figure 6:**
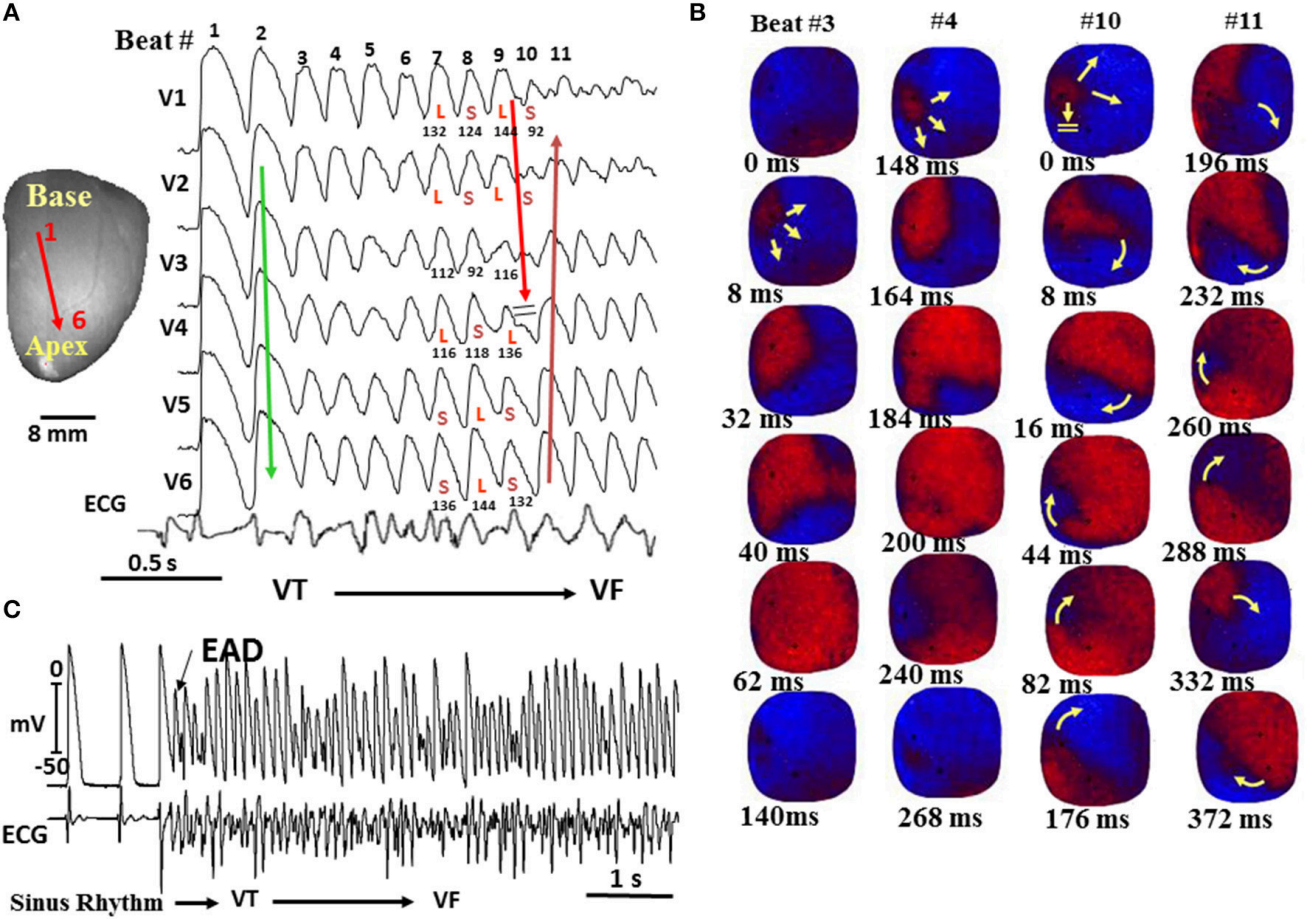
**Spontaneous initiation of VF in a middle-aged rabbit exposed to 0.1 mM H_**2**_O_**2**_. (A)** Six epicardial optical APs (V1–V6) recorded from sites shown on the left silhouette of the heart. After nine focal activations arising from the base of the heart **(B)**, the wavefront undergoes block at mid-LV anterior wall after spatially discordant APD alternans emerges **(A)**. The wavefront, however, continues to propagate lateral to the site of block in a clockwise direction causing a reentrant excitation as shown in the snapshots in **(B)**. The numbers under each snapshot is activation time starting with 0 ms (arbitrary) for beats 3 and 4 and then again for beats 10 and 11. **(C)** microelectrode recording of another VF episode recorded in the same heart showing spontaneous initiation of VF by a mechanism compatible with early afterdepolarization (EAD)-mediated triggered activity (TA) as in the aged rat hearts shown in Figure 6 (From Morita et al., 2009).

**Figure 7:**
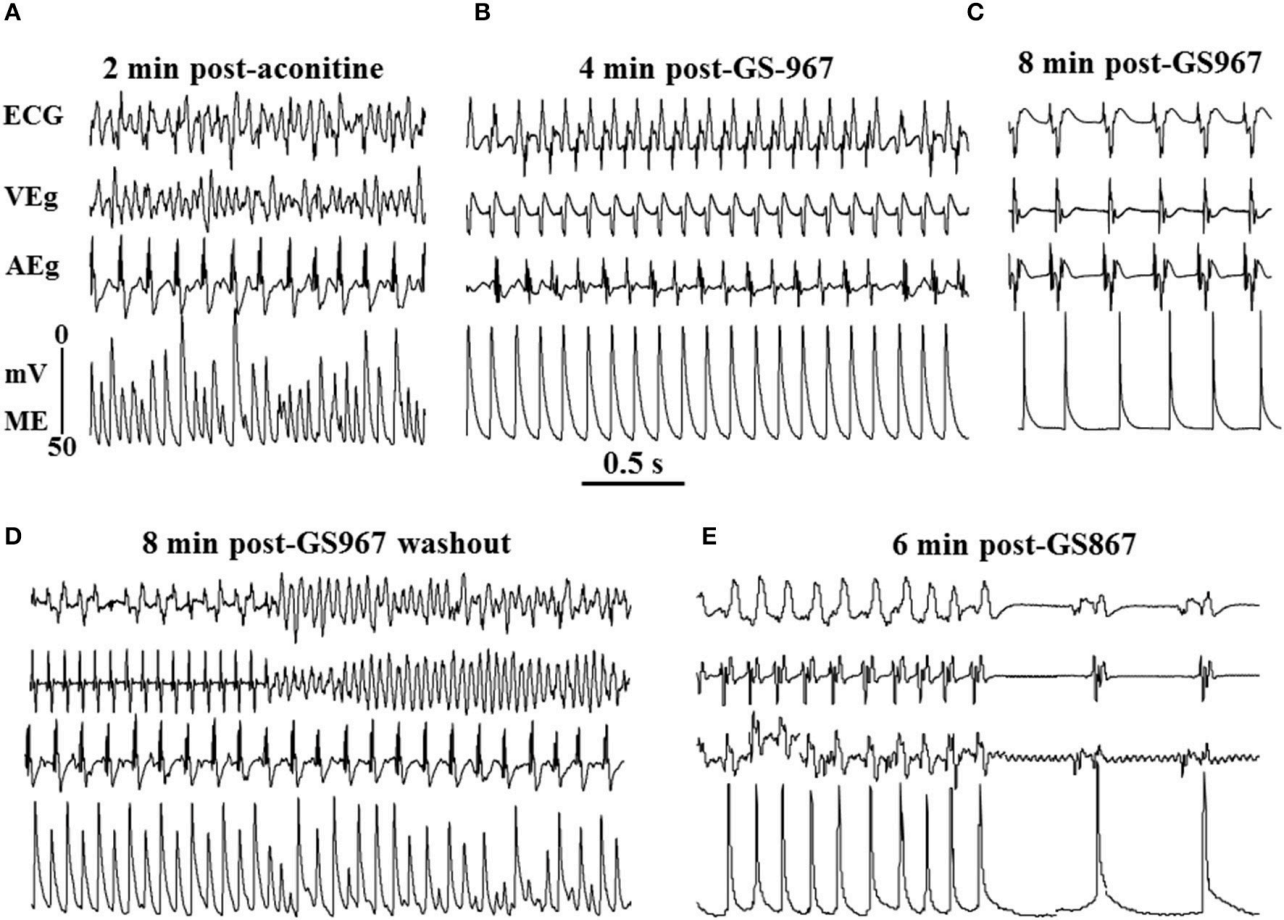
**Suppression of aconitine-induced ventricular tachycardia/ventricular fibrillation (VT/VF) with the specific I_**Na-L**_ blocker, GS967(1 μM) and reversal of its effect on washout. (A)** Aconitine-induced VF, which converts to monomorphic VT 3 min after arterial perfusion of GS967 **(B)** and to sinus rhythm 7 min after GS967 **(C)**. Washout of GS967 with drug-free Tyrode's perfusion causes the reemergence of monomorphic VT that degenerates to VF 8 min after GS967 washout **(D)**. The reintroduction of GS967 in the perfusate suppresses the VF by first converting it to monomorphic VT and then to sinus rhythm **(E)**. Note the marked shortening of the action potential duration after GS967-induced conversion of the monomorphic VT to sinus rhythm despite lengthening of the cycle length **(C, E)** (from Pezhouman et al., 2014b).

**Figure 8:**
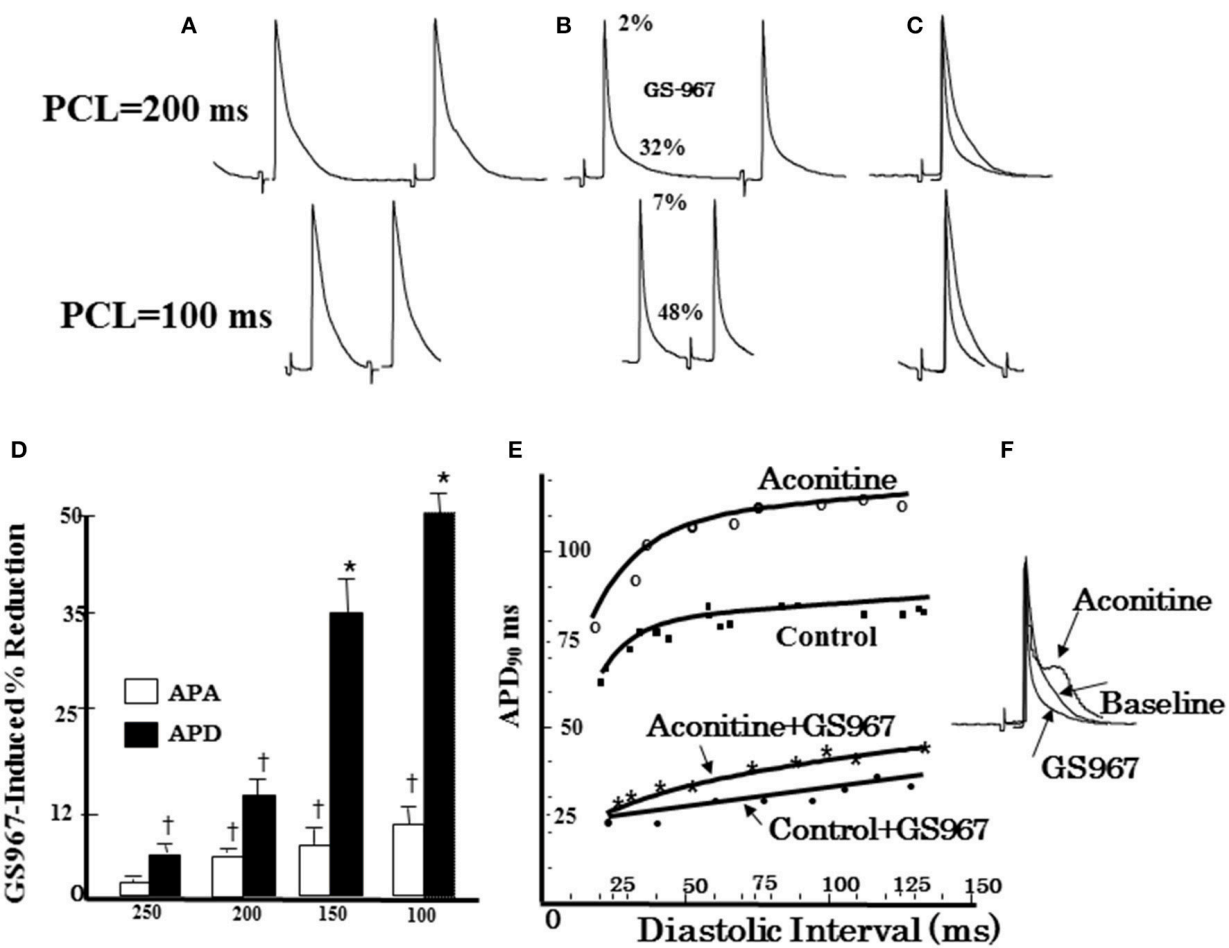
**Effect of GS967 on use-dependent action potential duration (APD), action potential amplitude (APA), and APD restitution. (A)** Baseline microelectrode recordings of action potentials at two pacing cycle lengths (PCLs): 200 ms (top) and100 ms (bottom). **(B)** Effects of GS967 at these two PCLs, causing 2 and 7% reduction in APA and 32 and 48% reduction in APD, respectively. **(C)** Superimposed APs showing that GS967 causes greater shortening of APD than APA at faster rates (use-dependent). **(D)** Mean percent shortening of APA and APD as a function of PCL. **(E)** Effect of GS967 on slope of dynamic APD restitution curves both at baseline and after aconitine. **(F)** Superimposed APs at baseline, after aconitine injection just before initiation of VT/VF and after GS967 (From Pezhouman et al., 2014b). ^*^p < 0.01; ^†^*p* < 0.05.

The EADs and the subsequent propagating triggered beats initiate diverse forms of ventricular arrhythmias starting from single PVCs, focal non-reentrant monomorphic or polymorphic VT and torsade de pointes that lead to the highly arrhythmogenic spatially discordant APD alternans causing wavebreak, reentry and transition from VT to VF (Morita et al., 2011a,b; Bapat et al., 2012; Chang et al., 2012; Pezhouman et al., 2015) as shown in Figures 5, 6. In addition to triggered foci, the oxidative stress may also initiate reentry by the phenomenon of “repolarization failure” during which the AP remains in the plateau region for a few seconds before undergoing full repolarization (Bapat et al., 2012). Localized myocardial regions with failed repolarization creates a considerable increase in spatial dispersion of repolarization promoting wavebreak and reentry (Weiss et al., 2000; Antzelevitch, 2008; Sato et al., 2009; Bapat et al., 2012; Karagueuzian et al., 2013). Heterogeneous increase in the late inward currents can also promote APD dispersion via their actions on the kinetics of APD restitution. Enhanced I_Na-L_ and late I_Ca,L_ by prolonging the APD increase the slope of the APD restitution curve (Qu et al., 2000; Guo et al., 2011; Morita et al., 2011a; Pezhouman et al., 2014b; Figure 7). Increasing the slope of the APD restitution curve promotes voltage- but not Ca-driven APD alternans (Weiss et al., 2006) which with a critical increase in the rate of activation converts the concordant APD alternans to the highly arrhythmogenic spatially discordant APD alternans (out-of-phase APD alternans) promoting unidirectional conduction block and reentry (Weiss et al., 2006; Morita et al., 2009; Karagueuzian et al., 2013). Selective block of the I_Na-L_ with GS-967 or selective block of the late I_Ca,L_ with roscovitine flatten the slope of the APD restitution curve preventing wavefront breakup and reentry by preventing the emergence of spatially discordant APD alternans (Pezhouman et al., 2014b, 2016; Angelini et al., 2016; Figures 7, 8). Interestingly flattening of the slope of the APD restitution with drugs that do not block the I_Na-L_ also prevent wavebreak and reentry but do not prevent EAD-mediated triggered activity (I_Na-L_ remains elevated) causing the maintenance of VT with no transition to VF. This dynamic scenario is confirmed with the studies with diacetyl monoxime (DAM), a drug that flattens the slope of the APD restitution curve without affecting the late currents (Coulombe et al., 1990; Riccio et al., 1999). DAM decreases in a concentration-dependent manner the amplitude of both the slow inward calcium current and the transient outward current, accelerating their inactivation and shifting their steady-state inactivation-voltage relationships toward negative potentials (Coulombe et al., 1990). In isolated-perfused swine ventricles in which EAD-mediated triggered VT/VF is initiated by myocardial local injection of aconitine (Swissa et al., 2002) an agent that selectively enhances the I_Na-L_ considerably (Peper and Trautwein, 1967), the administration of DAM prevented transition of aconitine-induced EAD-mediated triggered VT to VF which remained maintained by a single triggered focus maintaining a monomorphic VT (Swissa et al., 2002). The flattening of the slope of the APD restitution curve by DAM differs from that of GS-967 in that the latter unlike DAM by selectively blocking the I_Na-L_ suppresses both EAD-mediated triggered activity and reentry caused by wavebreak due to the flattening the slope of the APD restitution resulting in the conversion aconitine-induced VF to sinus rhythm (Pezhouman et al., 2014b; Figures 7, 8).

## Specific vs. non-specific block of drugs and VT/VF

**Figure 9:**
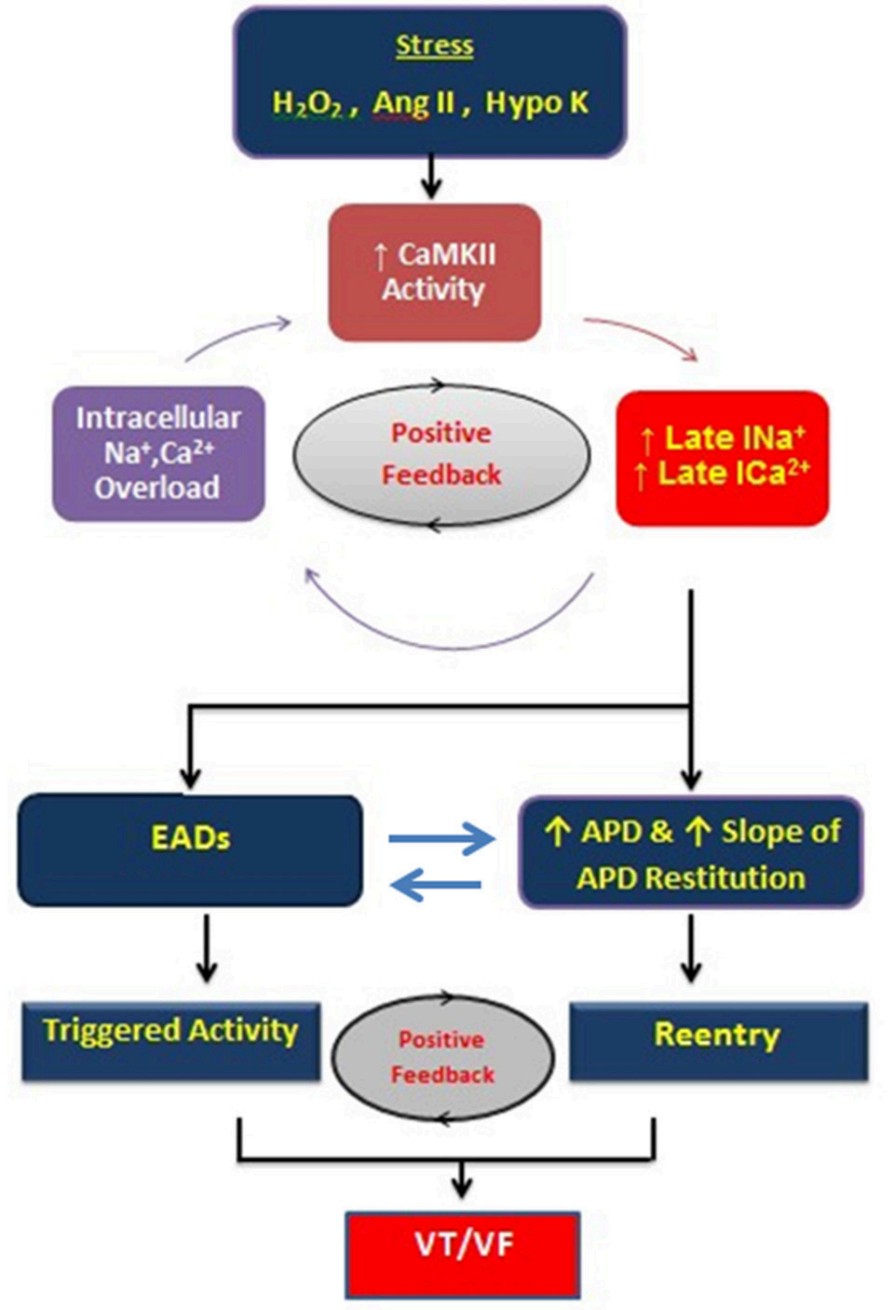
**Schema of positive feedback loops promoting intracellular Na and Ca overload, CaMKII activation, and EADs during stress induced with hydrogen peroxide, angiotensin II, and hypokalemia**. The enhanced late inward currents potentiate CaMKII activity (positive feedback loop) by prolonging APD and promoting EADs leading to VT/VF. APD indicates action potential duration; CaMKII, Ca-calmodulin kinase II; EAD, early afterdepolarization.

Many of the Class I AADs like mexiletine, flecainide, and propafenone, while blocking the peak I_Na_ (Figure 1) also manifest variable degree of block of the I_Na-L_ as well (Antzelevitch et al., 2014). However, the potential of proarrhythmia with these agents led to the development of more selective blockers of I_Na-L_, with no appreciable effect on the peak I_Na_ (Belardinelli et al., 2013). Furthermore, Class IC drugs like flecainide and propafenone in addition of being potent peak I_Na_ inhibitors also inhibit ryanodine 2 (RyR2) channels in the open-state, suppressing Ca^2+^ waves and preventing VT in the congenital catecholaminergic polymorphic ventricular tachycardia (CPVT) both in mice and humans (Hilliard et al., 2010). Clinical studies have shown that CaMKII activation in heart failure (Swaminathan et al., 2012) with its downstream increases of both I_Na-L_ and late I_Ca-L_ may require the block of both late inward currents to be effective against VT/VF (Figure 9). Support to this contention comes from simulation (Foteinou et al., 2015) and clinical studies that showed the need to combine ranolazine with verapamil to effectively suppress VT/VF in a patient with TS (LQT8) (Shah et al., 2012). While triggered activity either by EAD and subsequent emergent of DADs (Pezhouman et al., 2014b) remains a viable mechanism of VT/VF, the prevailing concept of AAD therapy in patients with healed myocardial infarction and other cardiomyopathies however, is largely based on how to disrupt reentrant excitation to terminate VT/VF (de Bakker et al., 1991; Pogwizd et al., 1992; Wu et al., 1998; Nash et al., 2006). Accordingly, the strategy of AAD is largely based (save for rare channelopathies, like CPVT) on a drug's ability to block ionic currents to disrupt reentrant arrhythmias. In fact, by directly altering cardiac wavefront conduction and refractoriness reentry may be prevented (Rosen and Janse, 2010). With this mindset it is proposed that Na channel blockers (i.e., Class I AAD) suppress reentry by decreasing conduction velocity and excitability converting unidirectional conduction block necessary for reentry formation to bidirectional conduction block preventing reentry and VT/VF (Karagueuzian and Chen, 2001). These expectations proved to be not only disappointing with respect to clinical therapy of VT/VF but also carried significant proarrhythmic risks. For example, the outcome of CAST clinical trial indicated that inhibition of peak Na current (Class 1C) was ineffective and even carried greater risk of mortality (Echt et al., 1991). Similarly, the SWORD clinical trials with d-sotalol (Class III) (Waldo et al., 1995) were also based on the premise of preventing reentry formation this time by increasing the cardiac refractoriness, an effect that will eliminate (close) the excitable gap necessary for reentry causing termination of the reentry. The results of CAST and SWORD showed drugs with Class I and Class III actions are ineffective in suppressing the arrhythmia following myocardial infarction (Echt et al., 1991; Waldo et al., 1995).

### New AAD class action (Class VI)

The current AAD classification, classically known as the Vaughan Williams classification, needs to be refined and updated with recent findings that attribute key arrhythmogenic role to the pathological rises of the late inward currents. As stated by Thireau and associates: “*The Vaughan Williams classification may no longer be best suited to the realities of new AA drug design”* (Thireau et al., 2011).

The key role played by the sustained elevation of the I_Na-L_ and late I_Ca,L_ in the genesis of focal and reentrant VT/VF qualify these pathological changes in ionic current kinetics *bone fide* “vulnerable parameters” of arrhythmias that are selectively targetable by small molecule drugs. In fact, the emergences of potent and prototype specific blockers of these late currents offers a new and effective AAD class action not considered previously. Since a fifth class of AAD actions is already assigned to diverse other agents (Table 1) it is suggested that the specific modifiers of the I_Na-L_ and late I_Ca,L_ (gating modifiers) be assigned a sixth AAD class action (Class VI). Prototype drugs with these class of actions are steadily becoming available including GS-967, the mexiletine analogs, HBRI21 & HBRI23 and roscovitine, that manifest high selectivity against the late I_Na-L_ and the I_Ca,L_ respectively without affecting their peaks (McKeithan et al., 2016). To distinguish between the two inward currents, the notations of Class VI_Na_ and Class VI_Ca_ may be added to indicate specific I_Na-L_ and late I_Ca,L_ blockers respectively. The concept of AAD therapy based on changes in ion channel gating rather than peak current block (i.e., gating modifiers) represents a turning point in AAD therapy that would provide a stimulus for future research in the field of AAD discovery that remained idle for decades (Kamath and Mittal, 2008; Thireau et al., 2011). Here, it is fitting to re-state the Editorial comments entitled “*Rational strategy to stop arrhythmias…”* made our group's findings (Madhvani et al., 2015) on the role of the late I_Ca,L_ in the genesis of EADs:

*“…Existing strategies for developing antiarrhythmic drugs have largely failed, and so new, innovative approaches as described by Madhvani et al. 2015 need to be aggressively pursued and tested” (Markandeya and Kamp, 2015)*.

## Author contributions

HK conceived and wrote the paper and RO discussed and added his recently published data and critically read the manuscript with suggestions and additions. AP and MA performed the experiments on the novel prototype drugs (Class VI), analyzed the data and prepared the figures for the manuscript.

## Funding

NIH, Supported in part by NIH/NHLBI grant P01 HL078931 and by UCLA Theodore Laubisch Endowment Fund.

### Conflict of interest statement

The authors declare that the research was conducted in the absence of any commercial or financial relationships that could be construed as a potential conflict of interest.

## Abbreviations

APD: action potential duration
AAD: antiarrhythmic drug
AF: atrial fibrillation
CaMKII: calcium/calmodulin-dependent protein kinase II
CPVT: catecholaminergic polymorphic ventricular tachycardia
CCB: calcium channel blocker
EAD: early after depolarization
DAD: delayed afterdepolarization
ECC: excitation–contraction coupling
VT: ventricular tachycardia
VF: ventricular fibrillation
I_Na-L_: late I_Na_
I_Ca,L_: L-type Ca current
VW: Vaughan Williams
LQT: long QT syndrome
RyR2: ryanodine receptor
PLB: phospholamban
SERCA2a: sarcoplasmic-endoplasmic Ca-activated ATPase pump
I_NCX_: Na-Ca exchanger current.
